# Using Plate-Wash PCR and High-Throughput Sequencing to Measure Cultivated Diversity for Natural Product Discovery Efforts

**DOI:** 10.3389/fmicb.2021.675798

**Published:** 2021-07-20

**Authors:** Emily N. Junkins, Bradley S. Stevenson

**Affiliations:** Department of Microbiology and Plant Biology, University of Oklahoma, Norman, OK, United States

**Keywords:** plate-wash PCR, cultivation, high-throughput sequencing, 16S rRNA gene sequencing, cultivation efficiency, road-kill, drug discovery

## Abstract

Molecular techniques continue to reveal a growing disparity between the immense diversity of microbial life and the small proportion that is in pure culture. The disparity, originally dubbed “the great plate count anomaly” by Staley and Konopka, has become even more vexing given our increased understanding of the importance of microbiomes to a host and the role of microorganisms in the vital biogeochemical functions of our biosphere. Searching for novel antimicrobial drug targets often focuses on screening a broad diversity of microorganisms. If diverse microorganisms are to be screened, they need to be cultivated. Recent innovative research has used molecular techniques to assess the efficacy of cultivation efforts, providing invaluable feedback to cultivation strategies for isolating targeted and/or novel microorganisms. Here, we aimed to determine the efficiency of cultivating representative microorganisms from a non-human, mammalian microbiome, identify those microorganisms, and determine the bioactivity of isolates. Sequence-based data indicated that around 57% of the ASVs detected in the original inoculum were cultivated in our experiments, but nearly 53% of the total ASVs that were present in our cultivation experiments were *not* detected in the original inoculum. In light of our controls, our data suggests that when molecular tools were used to characterize our cultivation efforts, they provided a more complete and more complex, understanding of which organisms were present compared to what was eventually detected during cultivation. Lastly, about 3% of the isolates collected from our cultivation experiments showed inhibitory bioactivity against an *already* multidrug-resistant pathogen panel, further highlighting the importance of informing and directing future cultivation efforts with molecular tools.

## Introduction

Molecular techniques and sequencing technologies have helped to describe many microbial communities and provide tools that allow us to study representative microorganisms and their activities in lieu of isolation. Application of molecular approaches to the study of microbiology have led to the discovery and characterization of rare biospheres of the most extreme environments (Sogin et al., 2006) and enabled researchers to decipher the metabolic potential and activity of microorganisms from the human gut (Donia et al., 2014) to the bottom of the ocean (Tortorella et al., 2018). Now, changes in microbial community structure and function can be monitored with molecular techniques throughout the course of a disease (Halfvarson et al., 2017) or with changing environmental conditions (Arora-Williams et al., 2018), such that we may begin to predict the roles of microbial populations and outcomes over time (Singh et al., 2010; Gilbert et al., 2016).

Isolating, or even just cultivating representative microorganisms is still considered the best means toward understanding their physiology, metabolism, and potential ecological roles, but this remains to be a major hurdle. The degree to which any microbial community is represented in culture varies considerably, though this has been the topic of debate (Martiny, 2019; Steen et al., 2019). Since Staley and Konopka coined the term “great plate count anomaly” (Staley and Konopka, 1985), numerous studies have sought to quantify the culturable organisms from various biomes [see (Lloyd et al., 2018), for a compiled list of most probable number (MPN) based approaches]. A recent study estimated that 81% of all microbial cells on Earth belong to uncultured genera or higher classifications and about 25% of microbial cells are from uncultured phyla (Lloyd et al., 2018). Specifically, 26% of marine, 31% of host-associated, 31% of eutrophic lakes, and 30% of soil metagenomic sequences have cultured representatives at the family level (Lloyd et al., 2018). Not surprisingly, human associated microbiomes have more cultured representatives, likely affected by a focused effort (Goodman et al., 2011; Eloe-Fadrosh et al., 2016; Lau et al., 2016). Sequencing technology has shed light onto the previously unknown diversity of the microbial world, outpacing our ability to cultivate these organisms by an estimated rate of 2.4- and 2.5-fold for bacteria and archaea, respectively (Schloss et al., 2016).

Efforts to isolate yet uncultured microorganisms have gathered momentum as researchers have begun to apply new technologies and innovations (Connon and Giovannoni, 2002; Stevenson et al., 2004; Nichols et al., 2010). Microbiologists routinely manipulate the composition of growth media and treatments of the inocula in order to increase the diversity of microorganisms represented in culture (e.g., Reasoner and Geldreich, 1985; Connon and Giovannoni, 2002; Davis et al., 2005; Kopke et al., 2005; Lagier et al., 2012; Oberhardt et al., 2015; Henson et al., 2016; Kawasaki and Kamagata, 2017; Kim et al., 2019; Bartelme et al., 2020). As an example, treating an inoculum with ethanol can select for spore-forming bacteria by killing vegetative cells and leaving spores intact that may otherwise be overgrown and remain unisolated (Browne et al., 2016). Furthermore, antioxidants in media can support the cultivation of aerobic and even anaerobic organisms, by alleviating oxidative stress (La Scola et al., 2014; Dione et al., 2016). The goals of this study were to (1) determine the efficiency of cultivating representative microorganisms from a non-human, mammalian microbiome, (2) identify cultivated microorganisms through 16S rRNA gene sequencing, and (3) screen representative isolates for antimicrobial activity. With one aim of this study being to cultivate many, different microorganisms, the approaches described above were of particular interest. We anticipated that we would select for gut-associated bacteria; antioxidants would help mitigate oxygen stress for organisms best suited for microoxic or anoxic environments, and ethanol treatment would reduce the abundant vegetative cells, allowing us to better access the many spore-forming taxa that reside in the gut.

When cultivation approaches are coupled with molecular analyses, the total diversity and identity of cultivated taxa can be determined (Yang et al., 2015; Medina et al., 2017; Justice et al., 2017), and specific, targeted taxa can be detected (Stevenson et al., 2004). More recently, high throughput sequencing has made it possible to quantify exactly what proportion of an original sample has been cultivated (Medina et al., 2017; Zehavi et al., 2018; Cummings et al., 2020). Studies using this approach showed that roughly 23% of microorganisms in bovine rumen fluid were cultivable in liquid enrichments, some of which were not detected in direct sequencing efforts (Zehavi et al., 2018), while 96% of those in human bronchoalveolar lavage samples (Cummings et al., 2020), and 60% of those detected in/on American toads (Medina et al., 2017) were cultivated. A recent study characterized the microbial diversity within the same inoculum spread onto both high- and low-nutrient media by harvesting biomass on each plate and identifying all taxa present (Medina et al., 2017). Their approach built upon a previous study that sought to isolate abundant but yet-uncultured taxa using defined, low nutrient media and a PCR-based screening approach using group specific primers, termed plate-wash PCR (PWPCR) (Stevenson et al., 2004). Medina et al. confirmed that low nutrient-media would allow for the growth of a more diverse subset of bacteria from the original sample and that molecular screening (i.e., 16S PWPCR) is a practical way to characterize the microbial community growing on an entire agar plate (Medina et al., 2017). This data is invaluable in identifying medium composition, inoculum treatments, and incubation conditions that can target certain taxa, or increase the cultivated diversity for large-scale isolation efforts (see Henson et al., 2016; Bartelme et al., 2020). However, these studies using solid agar plates may have overestimated diversity for some taxa by focusing on the relative abundance of taxa in their analyses.

Pure cultures have always been the “gold standard” for the in-depth study of microorganisms. The need for isolation is especially important when screening for the production of secondary metabolites for drug discovery. Historically, the process of isolating microorganisms for drug discovery has relied mainly on high throughput cultivation efforts that increase the number of isolates being screened (e.g., Motley et al., 2017; Helfrich et al., 2018; Hoffmann et al., 2018), enrich for bioactive production from known producers like *Streptomycetes* spp. (van der Meij et al., 2017), or focus on novel or rare taxa thought to have uncharacterized biosynthetic capabilities (Stierle et al., 2006; Ding et al., 2010; Rangseekaew and Pathom-aree, 2019). We aimed to do the same by sampling a host-associated microbial community and cultivating them under conditions designed to obtain as many different organisms as possible through the use of different media types. Screening cultivated microorganisms with the broadest diversity is expected to provide the greatest chance of uncovering natural products that are novel drug targets. Previous studies have focused on extreme environments for novel natural products (Smanski et al., 2015), but we expand on this idea to include non-human mammals. We predicted that this would increase the diversity of microorganisms and compounds screened for useful antimicrobial compounds and argue that the shared life-history between a host and members of its microbiomes may produce compounds that are less cytotoxic to the host. This idea was originally supported by a survey of microbiomes collected from roadkill mammals, which produced two new cyclic lipodepsipeptides of pharmaceutical interest (Motley et al., 2017).

Here, we sought to use both general and selective media along with various treatments to the inocula to collectively enrich for as many different microorganisms as possible from the oral and gut microbiomes of a roadkill mammal (raccoon). The goals for this study were to (1) compare the diversity of cultured bacterial communities on multiple types of media combined with various treatments of the inoculum, (2) determine which media, treatments, or combination cultivated the highest richness of bacterial taxa, and (3) determine how many isolates produced bioactive molecules against a panel of multi-drug resistant pathogenic microorganisms.

## Materials and Methods

### Sample Collection and Storage

A 3 mile stretch of Oklahoma State Highway 9 in Norman, OK between the University of Oklahoma, Norman campus and Lake Thunderbird was used for our opportunistic sampling area. This well-traveled highway runs through undeveloped prairie, scrub and forest, cattle ranches, and rural residential land, providing many mammalian roadkill specimens. Sample collection was conducted under Oklahoma Scientific Collector Permit #5250 (Motley et al., 2017). The oral cavity and rectum of a raccoon (*Procyon lotor*) deceased less than 8 h was sampled in triplicate (*n* = 3) with sterile, nylon swabs. One concern was that swabbing for sequencing first would remove or alter the biomass when the cultivation swab samples were taken. To avoid this, each orifice was swabbed three times. At each of those times, two swabs were used, one destined for cultivation the other destined for sequencing. In doing so, any alteration of the biomass would be reflected in each set of triplicate swabs, for sequencing and for cultivation. This generated a total of 6 swabs from each orifice, 3 for sequencing and 3 for cultivation. Swabs to be used for cultivation were pooled by orifice and stored in a sterile conical tube containing 3.0 mL 1X PBS, stored on ice, and immediately transferred back to the laboratory (under 45 min). Swabs to be used for sequencing were transferred to separate, empty, sterile conical tubes to preserve the technical replicate, stored on ice, and immediately transferred back to the laboratory (under 45 min). At the laboratory, swabs for sequencing were transferred to BashingBead Lysis Tubes (Zymo Research Corp., Irvine, CA) containing 750 mL of BashingBead Buffer (Zymo Research Corp.). Cells were lysed through homogenization in a Mini-BeadBeater-8 (BioSpec Products Inc., Bartlesville, OK) at maximum speed for 45 s. Homogenized samples were then stored at −20°C until needed for DNA extraction. Swabs for cultivation were vortexed to suspend cells. A 900 μL aliquot of the cell suspension intended for storage was centrifuged at 10,000 × g for 1 min, the supernatant was removed. The cell pellet was resuspended in 750 mL of BashingBead Buffer (Zymo Research Corp.) and transferred to BashingBead Lysis Tubes (Zymo Research Corp., Irvine, CA) prior to lysis through homogenization in a Mini-BeadBeater-8 (BioSpec Products Inc., Bartlesville, OK) at maximum speed for 45 s. Archives were stored at −80°C. The remaining 2.1 mL of the cell suspension for each sample was serially diluted with PBS. Aliquots of 50 μL from the 10^0^–10^–6^ dilutions were spread onto 13 different media/treatment combinations (see Supplementary Table 1). In all, each inoculum was spread onto the agar media, n = 6, for each medium type and incubated for 6 days at 30°C, three plates would be used for PWPCR and three plates would be used to pick colonies for isolation. As a control, 50 μL of PBS used for sample collecting and dilutions was spread onto each medium type and incubated for 6 days at 30°C.

### Culture-Dependent Bacterial Community Analysis

Cultivation was conducted with a combination of broad-spectrum media with decreased nutrients and selective media in order to “cast a wide net” and increase the cultivable diversity of each inoculum (see quasi-factorial design in Supplementary Table 1). The media used included 0.1X strength tryptic soy broth (Bacto, United States) with 1.5% agar, ROXY and derivatives with hemin (0.1 g/L) and alpha-ketoglutarate (2.0 g/L) (referred to as ROXYakgh) (Dione et al., 2016), 0.25X strength R2A medium (Reasoner and Geldreich, 1985), and blood agar (per L: 10.0 g meat extract, 10.0 g peptone, 5.0 g sodium chloride, 15 g agar, 5% sheep’s blood (Thermo Fisher Scientific, United States), pH 7). To date, this is the first instance using ROXY medium and plate-wash PCR techniques. In addition to different media types, one variation included the pretreatment of the inocula for 4 h in 70% ethanol at 22–24°C (Browne et al., 2016). Catalase (40,000 U/L) was added as a treatment to TSA, ROXY, and R2A to remove peroxides produced during autoclaving (Kawasaki and Kamagata, 2017; Kim et al., 2019). The last treatment included the addition of streptomycin at 50 mg/L as a broad spectrum selection agent against Gram-negative and some Gram-positive species (Schatz et al., 1944). To test which combinations of media and treatments resulted in cultivated communities that represented the diversity observed in the original sample, we grew each inoculum (mouth or rectum) on each combination of media/treatment types and sequenced the resulting biomass.

### Collection of Total Biomass From Agar Plates, DNA Extraction and PCR (PWPCR)

Biomass was harvested from each agar plate in order to identify the microorganisms present and to compare this to culture-independent analyses of the inoculum. The biomass from each agar plate was harvested by adding 2.0 mL of PBS to the surface of the plate and suspending the colony biomass by agitation with a sterile spreader. The suspended colony biomass was collected and transferred to a 2.0 mL microcentrifuge tube, pelleted at 10,000 × g for 1 min, and resuspended in 750 μL of BashingBead Buffer (Zymo Research Corp.). Each sample was transferred to a BashingBead Lysis Tube (Zymo Research Corp.) and homogenized for 45 s at maximum speed using a Mini-BeadBeater-8 (BioSpec Products Inc., Bartlesville, OK). The lysed samples were stored at −20°C until DNA was extracted.

### DNA Extraction and Sequencing

Before DNA extraction, each sample was thawed on ice and homogenized for 30 s at maximum speed using a Mini-BeadBeater-8 (BioSpec Products Inc., Bartlesville, OK). DNA was extracted according to manufacturer specifications using the Zymo *Quick*-DNA Fungal/Bacterial Miniprep kit (Cat# D6005, Zymo Research Corp., Irvine, CA). For community characterization, a conserved region (V4-V5) of the SSU rRNA gene of most bacteria, archaea, and eukarya was amplified using primers 515F-Y and 926R (Parada et al., 2015) via the following PCR protocol: initial denaturation at 94°C for 2 min, followed by 30 cycles of denaturation at 94°C for 45 s, annealing at 50°C for 45 s, and extension at 68°C for 90 s, with a final extension at 68°C for 5 min. These primers produced two amplicons, a ∼400 bp fragment for bacteria and archaea, and a 600 bp fragment for eukaryotes. The forward primer 515F-Y (5′-GTA AAA CGA CGG CCA G CCG TGY CAG CMG CCG CGG TAA-3′) contains the M13 tag (underlined) fused to the 5′ end of the forward primer (Kraus et al., 2018). The reverse primer 926R (5′-CCG YCA ATT YMT TTR AGT TT-3′) was unmodified from Parada et al. (2015). Each PCR contained 5 PRIME HOT master mix (1X; 5 PRIME Inc., Gaithersburg, MD), 0.2 μM of each primer, and 3.0 μL of extracted DNA at a final volume of 50 μL. The amplified fragments were purified using Sera-Mag magnetic beads (GE) with the AmPureXP (Beckman Coulter) protocol at a final concentration of 1.8x v/v. After purification, 3 μL of each PCR product, 1 × 5 PRIME HOT master mix (Quantabio, Massachusetts, United States), 0.2 μM of the 926R primer, and 0.2 μM of a specific 12 bp oligonucleotide was used in a separate barcoding PCR (6 cycles) in 50 μL reactions to attach a unique barcode to amplicons of each library. The same thermocycler protocol was used as above but only run for 6 cycles. The now barcoded amplicons were purified using Sera-Mag (GE) beads with the AmPureXP (Beckman Coulter) protocol to a final volume of 40 μL, quantified using the QuBit HS DS DNA assay kit (Thermo Fisher Scientific Inc., Waltham, MA), and pooled in equimolar amounts. The pooled, barcoded amplicon libraries were then concentrated to a final volume of 40 μL (209 ng/μL) with an Amicon-Ultra filter (Millipore, Burlington, MA, United States) following manufacturer’s protocol. The combined amplicon libraries were denatured according to Miseq library preparations protocol (Illumina, San Diego, CA, United States). The sample was loaded at a concentration of 10 pM and sequenced using 2 × 250 paired-end strategy on the Miseq (Illumina San Diego, CA, United States) platform for 251 cycles.

### ASV Inference and Bacterial Community Characterization

Barcodes from amplified regions of the 16S rRNA gene were removed and demultiplexed using QIIME v 1.9.1 (Caporaso et al., 2010). Demultiplexed reads were trimmed for adapters and quality filtered as a part of the *dada2* pipeline (Callahan et al., 2016) and amplicon sequence variants (ASVs) were inferred using the forward reads (203 bp). Specifically, forward reads were used instead of merged forward and reverse reads due to poor quality in the overlap region. In order to retain sequencing depth, but at the expense of some taxonomic resolution, reverse reads were omitted from all analyses. In short, forward reads (V4) were trimmed and quality filtered (maxEE = 2) via *filterAndTrim*, depreplicated via *derepFastq*, and ASVs were inferred (*dada*). Concerning ASV filtration, the dada2 pipeline does not infer an amplicon sequence variant that is only supported by one read, therefore, the final ASV table does not contain singletons, which could affect statistical methods for richness (though no statistics were performed on richness in this study). Lastly, chimeras were removed (*removeBimeraDenovo*, method = “consensus”) and taxonomy was assigned using the SILVA database v32 (Quast et al., 2013; Yilmaz et al., 2014). Sequences mapping to chloroplasts and mitochondria were filtered from the dataset. The final dataset consisted of ∼1.7 million reads resulting in 463 ASVs with a median of 17,347 sequences per sample (min = 132, max = 266,678).

#### Data Availability

Sequence data has been deposited at NCBI’s Sequence Read Archive (SRA) database under accession number PRJNA675861.

### Data Analysis

Community diversity was analyzed using the *phyloseq* (McMurdie and Holmes, 2013) and *vegan* (Dixon, 2003) packages in R. Relative abundance was only used to directly assess the diversity of the original inocula. Presence/absence and richness were used as the main metrics to compare cultivated samples because of the variation in biomass (i.e., colony size) between microbial colonies on and between agar plates. Since this procedure duplicated swabs when sampling, the data from the molecular control swabs were pooled for analysis. The variability in swabbing, as these are technical replicates, would be different from the variability of triplicates resulting from cultivation, since these could represent biological replicates resulting from the stochasticity of what cells landed on which plate.

Alpha diversity was calculated as richness of ASVs, while beta diversity was measured with non-metric multi-dimensional scaling (NMDS) using Jaccard distances. A permanova with the *adonis2* function in the *vegan* package was used to compare differences in the composition of taxa cultivated across media types, treatment, and orifices (Dixon, 2003). Due to the nestedness of orifice and cultivation conditions, orifice was used as a strata or fixed factor, and media and treatment were used as predictor variables (∼media^∗^treatment). Differences in cultivable diversity could be due to different starting communities from each orifice. So, orifice was treated as a fixed factor to control for any influence it could have on the significant differences in diversity between cultivable communities. To fully understand the significance described in the permanova, a permdisp with the *betadisper* function in *vegan* was used to describe any within sample variance (i.e., across replicate agar plates) that could explain any significant differences detected in the permanova. Hypothesis testing via ANOVA with permutations (*n* = 999) was used with permdisp to determine any significant differences in variation within samples. These statistical methods allowed us to determine if our cultivation strategies cultivated diverse subsets of organisms that were different from each other between media and treatment types.

To identify certain ASV families more associated with an orifice, medium, or treatment, a species indicator analysis was performed using the *indicspecies* package (permutations = 999) (De Cáceres and Legendre, 2009; De Cáceres et al., 2011). Species indicator analyses would help efforts to determine which medium or treatment cultivated which taxa.

### Bioassays and Isolate Identification

Colonies were selected for isolation based on morphology, with the intent of sampling as many different colony morphologies as possible. From average of 8.1 × 10^10^ CFUs/mL cultivated across all medium types and dilutions, a total 238 colonies were chosen for isolation based on differential colony morphology from the mouth and rectum on a subset of the media types (0.1X TSA, ROXY with alpha-ketoglutarate and hemin, and 0.25X R2A). Colonies were isolated by streaking for isolation of single colony morphologies at least three times before the assumption of pure cultures. To determine bioactivity, soft agar overlays in 0.1X TSA (0.8% agar) were used to test for growth inhibition of a panel of drug-resistant pathogens (*Klebsiella pneumonia* ATCC# 13883, *Enterococcus faecium* ATCC# 51559, *Pseudomonas aeruginosa* ATCC# 10145, and *Candida albicans* ATCC# 565304). To prepare for the bioassay, pathogens were incubated overnight, shaking at 250 rpm at 30°C, in 0.1X tryptic soy broth (TSB) (Bacto, United States). A 400 μL aliquot of each pathogen was used to inoculate 3.6 mL of molten TSA soft agar (at 55°C), mixed, and immediately poured onto a 0.1X TSA agar plate for a final overlay volume of 4 mL. Colony material from each isolate was transferred to the bioassay plate with a sterile toothpick by etching an “X” into the overlay. The inoculated bioassay plates were incubated at 30°C for 24 h. Inhibition of the pathogen in the overlay was characterized by a zone of clearing surrounding the isolate (Supplementary Figure 1). For cryopreservation, each isolate was grown in 5 mL of 0.1X TSB in a 16 × 150 mm test tube shaking at 250 rpm for 48 h. An aliquot of 800 μL of the culture was transferred to a 2 mL screw cap tube, along with 200 μL of 80% glycerol, for a final concentration of 20% glycerol. After mixing by vortex for 30 s, it was then stored at −80°C.

Partial SSU rRNA gene sequences were used to identify each isolate. Genomic DNA was extracted from each isolate using QuickExtract (Lucigen, Wisconsin, United States) according to manufacturer’s protocol from the above mentioned 0.1X TSB cultures. The SSU rRNA gene was amplified using primers 8F (3′-ATGC-5′) and 1492R (3′-ATGC-5′) at a concentration of 0.2 mM, 1 × 5 PRIME HOT master mix (Quantabio, Massachusetts, United States), and 2.0 mL of DNA template per 50 mL reaction via the following PCR protocol: initial denaturation at 94°C for 2 min, followed by 30 cycles of denaturation at 94°C for 30 s, annealing at 55°C for 45 s, and extension at 72°C for 45 s, with a final extension at 72°C for 10 min. Amplified fragments were purified using Sera-Mag magnetic beads (GE) with the AmPureXP (Beckman Coulter) protocol at a final concentration of 1.8x v/v. Some isolates were not recoverable after the process of freeze-thawing, and some extractions did not result in enough DNA for sequencing. Purified amplicons (a total of 192 samples) were sent for Sanger sequencing using the 8F primer (Genewiz, New Jersey, United States). Resulting sequences and chromatograms were assessed for quality using Integrative Genomics Viewer (IGV) (Robinson et al., 2011). Sequences with low quality (<Q20) were removed, low quality ends were trimmed with MEGA X (Kumar et al., 2018), and the final sequences were identified using the SILVA v. 132 classifier online server (Quast et al., 2013). A total of 117 isolates had suitable reads and met quality thresholds for taxonomic identification.

## Results

### Cultivated Microbial Richness Differed Between Orifice and Cultivation Condition

**FIGURE 1:**
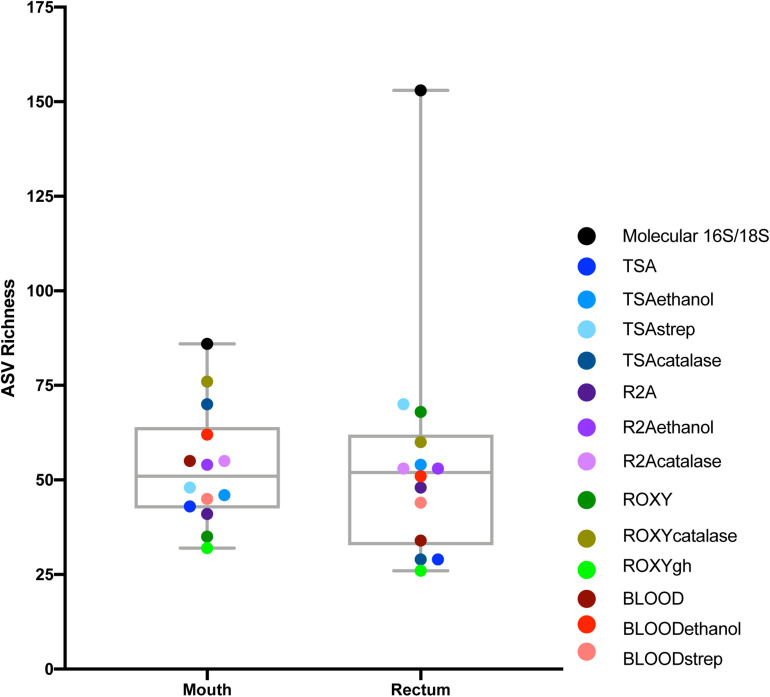
Alpha diversity of the mouth and rectum. For the controls, richness, measured using ASVs, was higher in the rectum than the mouth. For cultivated communities, richness varied based on medium type and treatment. Points represent each medium or treatment type, indicated by color (*n* = 3). Molecular 16S/18S refers to the directly sequenced inoculum sample (*n* = 3).

**FIGURE 2:**
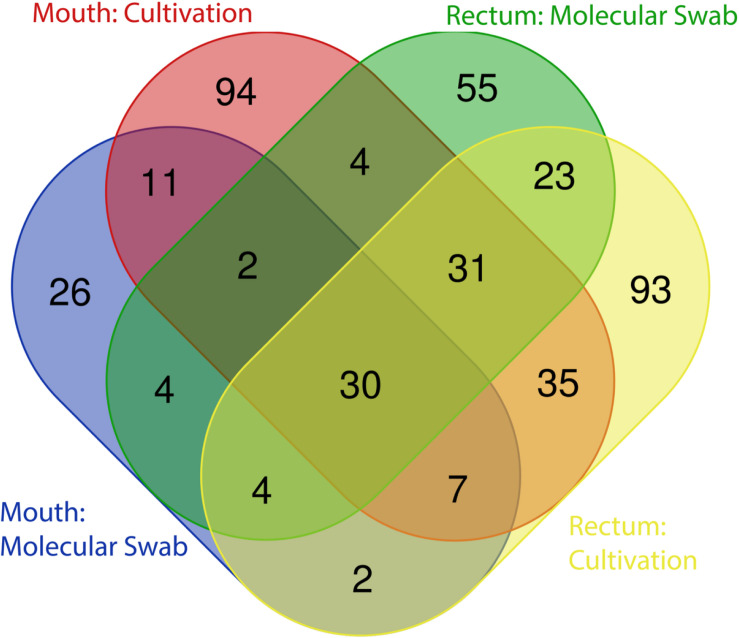
Distribution of ASVs between cultivation and molecular controls. The majority of ASVs detected in the original inocula were shared between the mouth and rectum, while approximately half of all ASVs detected through cultivation were not detected in the directly sequenced samples.

**FIGURE 3:**
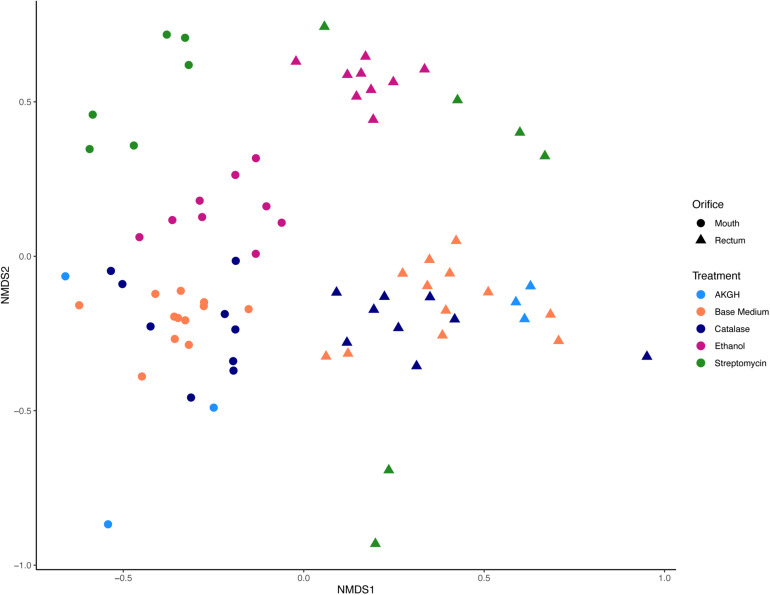
Beta diversity measured in Jaccard distance and NMDS ordination. Differences in microbial community structure were driven by orifice and treatment, specifically ethanol and streptomycin. Shape corresponds to orifice sampled, while color differentiates between treatments of the medium or inoculum.

As expected, a library of SSU rRNA genes from the original inocula showed that they had higher richness (alpha diversity) than the cultivated organisms from both orifices (Figure 1). The microbial community sampled from the rectum had higher overall richness (153 ASVs) compared to that of the mouth (86 ASVs) (Figure 1). Overall, we were able to cultivate 57.3% of the ASVs detected in the inocula (58.1% from the mouth and 57.5% from the rectum). Interestingly, about half (52.7%) of the ASVs detected from the cultivation experiments were not represented in either library from the inocula (i.e., molecular control) (Figure 2). The number of ASVs detected on a certain medium or treatment differed between orifices. For instance, cultivation on TSA medium treated with catalase resulted in only 29 ASVs from the rectum inoculum compared to around 70 different ASVs from the mouth. The fewest number of ASVs was detected on the ROXY medium amended with alpha-ketoglutarate and hemin from both the mouth and rectum. The cultivated beta diversity differed based on orifice and cultivation condition. Specifically, community structure differed based on orifice and treatments, more so than base medium type (Figure 3). Differences in the cultivated community composition and diversity between medium and treatment confirmed that, collectively, using different cultivation techniques increased the number of organisms cultivated from each inoculum.

### Selective Treatments Increased Cultivated Richness Compared to Medium Type or Orifice

An indicator species analysis was used to determine which microbial families were significantly associated with certain conditions. A total of ten families were identified to be significantly (*p* < 0.05) associated with orifice, medium type, and/or treatment. Only one family, the Aeromonadaceae, was significantly associated with a particular orifice, the mouth. This family was also the only family significantly (*p* = 0.001) associated with a certain base medium type, ROXY (Table 1). Treatment of the medium and/or the inoculum selected for the most diverse set of indicator species, with a total of 9 families significantly (*p* < 0.05) associated with a certain treatment or combination of treatments (Table 1). Certain bacterial families like Staphylococcaceae and Paenibacillaceae were associated with the ethanol pretreatment of inocula, whereas the Wohlfarhtiimonodaceae and Metaschinikowiceae were more associated with the addition of streptomycin to media (Table 1).

**TABLE 1:** Taxonomic associations with media type using indicator species analysis.

**Grouping factor**	**Condition**	**Indicator ASV family**	**Index value**	***p*-value**
**Orifice**	Mouth	Aeromonadaceae	0.483	0.007
**Media type**	ROXY	Aeromonadaceae	0.589	0.001
	Blood + TSA + R2A	Cladosporiaceae	0.604	0.026
**Treatment**	Ethanol	Staphylococcaceae	0.562	0.014
		Paenibacillaceae	0.408	0.041
	Streptomycin	Wohlfahrtiimonadaceae	0.408	0.034
		Metschnikowiaceae	0.408	0.030
	Ethanol + Streptomycin	Peptostreptococcaceae	0.669	0.003
	Catalase + Ethanol + Streptomycin	Aspergillaceae	0.692	0.004
		Cladosporiaceae	0.633	0.006
	Catalase + Ethanol + Streptomycin + No Treatment	Debaryomycetaceae	0.899	0.002
		Clostridiaceae 1	0.860	0.006

**FIGURE 4:**
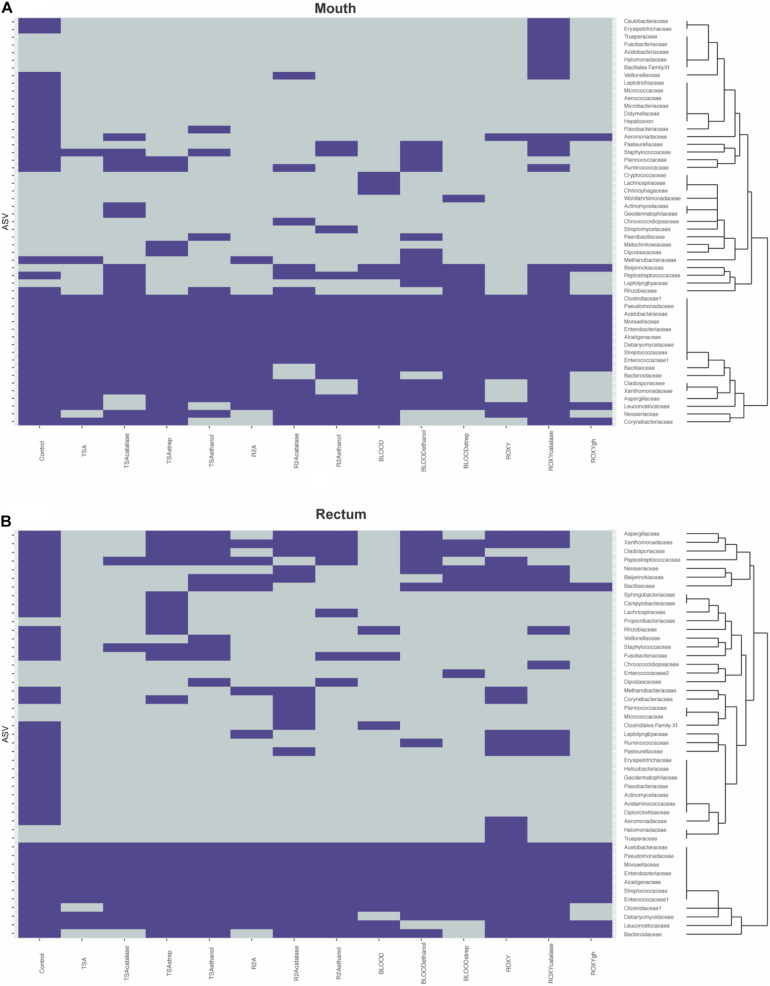
Heatmaps describing presence absence of taxonomic families observed in the **(A)** mouth and **(B)** rectum. Purple indicates that a family was observed while gray indicates that a family was absent. The dendrograms highlight clusters of families observed during cultivation. Control refers to the families identified on the molecular control.

Much of the cultivable microbial diversity was shared between the mouth and the rectum, indicated by the lack of significant differences in community structure described by the permanova and permdisp analyses (discussed below). Based on molecular analyses, 27 families were shared between the mouth and rectum before cultivation, while 6 families were unique to the mouth and 6 families were unique to the rectum (Figure 4). Negative cultivation controls (e.g., PBS) yielded no growth, supporting the assumption that any biomass collected from plates originated from the inoculum. Lastly, our cultivation experiments led to the detection of 18 families from the mouth and 8 families from the rectum that were not detected through direct molecular analysis of the inoculum (Figure 4). This accounted for around half (52.7%) of the ASVs detected during cultivation (Figure 2). This could be explained by differences in biomass and growth characteristics not being captured by sequence data from the inocula (discussed below).

### The Raccoon Microbiome Contained Cultivable Bioactive Bacteria

A total of 238 isolates were collected from a subset of media (0.25X R2A, 0.1X TSA, and ROXY with alpha-ketoglutarate and hemin). Despite choosing colonies based on differential morphology, many of these isolates were expected to be redundant, since the diversity of the initial inoculum was low (Figure 1). This was confirmed with many isolates redundant at the genus level and the majority of the identifiable isolates belonging to genera *Serratia* (34.2%) and *Klebsiella* (17.9%) (Supplementary Figure 2). Each of the 238 isolates were assayed for antimicrobial activity against a panel of *multi-drug resistant* pathogens. A total of 7 isolates showed antimicrobial activity, identified as a zone of clearing of one of the antibiotic resistant panel organisms. This equated to a ∼3% “hit” rate against already resistant organisms. Six of these isolates were recovered from the mouth, while one, a *Bacillus* sp., was recovered from the rectum. Three isolates were recovered from R2A, 3 from TSA and 1, an *Enterobacteriaceae* sp., from ROXYakgh. Three isolates exhibited antimicrobial activity against *Klebsiella pneumonia*, three against *Enterococcus faecium*, and one, a *Pseudomonas* sp., against *Candida albicans.* Five of the isolates were identified by partial 16S rRNA gene sequence identity (Table 2).

**TABLE 2:** Cultivated bioactive isolates.

**Isolate**	**SILVA taxonomy**	**Orifice**	**Medium**	**Activity against**
RC2RCR2A30 -212	*Bacillus* sp.	Rectum	R2A	*Klebsiella pneumoniae*
RC2MOR2A30 -238	*Pseudomonas* sp.	Mouth	R2A	*Candida albicans*
RC2MORGH30 -018	Enterobacteriaceae	Mouth	ROXYakgh	*Enterococcus faecium*
RC2MOTSA30 -050	*Enterococcus* sp.	Mouth	TSA	*Enterococcus faecium*
RC2MOTSA30 -142	*Klebsiella* sp.	Mouth	TSA	*Klebsiella pneumoniae*

### The Composition of Cultivated Taxa Was Highly Variable

**FIGURE 5:**
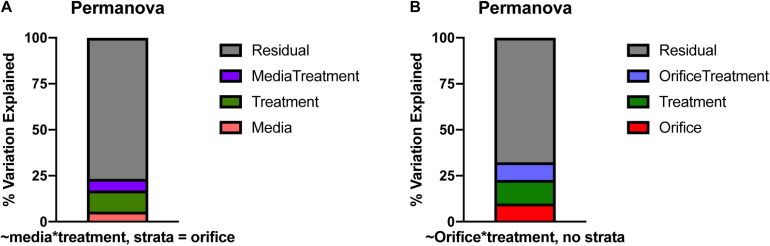
Percent of variation described by permanova models based on **(A)** media and treatment with orifice as a stratum, **(B)** orifice and treatment. Largely, the differences in community structure during cultivation could not be explained by media, orifice, or treatment alone. In all graphs, residual variation (in gray), is the proportion of variance not explained by the model.

Due to the nestedness of this experiment (i.e., taxa observed during cultivation theoretically would be observed in sequencing), the stratification of taxa based on sampled orifice was corrected for hypothesis testing with permanova by defining orifice as a stratum in the *adonis2* function in the *vegan* package (Dixon, 2003; Figure 5A). The majority of the explained variation and significant differences in cultivated taxa were due to treatment of the inoculm or medium type (*F* = 2.44, *R*^2^ = 0.12, *p* = 0.001), while base medium type alone explained only ∼5% of the observed variation (*F* = 1.54, *R*^2^ = 0.05, *p* = 0.002). The interactions of base medium and treatment were insignificant and did not fit the model (*F* = 1.52, *R*^2^ = 0.06, *p* = 0.208). An analysis of variances (permdisp) was used to determine if any significance was driven by a dispersion effect rather than a location effect for orifice, base medium type, treatments, and medium + treatments. Mean distances to centroids did not differ significantly for orifice (*F* = 0.69, *p* = 0.41; Mouth 0.51; Rectum 0.53) or medium + treatment (*F* = 1.38, *p* = 0.20; TSA = 0.47, TSAcatalase = 0.52, TSAstrep = 0.51, TSAethanol = 0.45, R2A = 0.44, R2Acatalase = 0.47, R2Aethanol = 0.45, Blood = 0.45, Bloodethanol = 0.50, Bloodstrep = 0.53, ROXY = 0.48, ROXYcatalase = 0.49, ROXYakgh = 0.52), meaning little inter-sample variation was detected between replicates. In contrast, the mean distances did differ for treatment (*F* = 3.3, *p* = 0.02; alpha-ketoglutarate + hemin = 0.52, no treatment (base) = 0.50, catalase = 0.53, ethanol = 0.49, streptomycin = 0.56) and base medium type (*F* = 4.4, *p* = 0.01; Blood = 0.56, R2A = 0.50, ROXY = 0.53, TSA = 0.55). The significance in dispersion within the treatment and media could correspond to the significance in permanova results, where much of the variation explained in the permanova may be due to the intrinsic variability and stochasticity among replicates of the same inoculum.

## Discussion

We used mouth and rectal samples from a roadkill mammal (raccoon, *Procyon lotor*) to (1) compare the diversity of cultured bacterial communities on multiple media types and/or treatments of the inoculum, (2) determine which media/treatments cultivated the highest richness of bacterial taxa, and (3) determine if any isolated organisms produced bioactive molecules against a *drug resistant* pathogen panel. The occurrence of antimicrobial resistance is increasing at a faster rate than new therapies are entering the market (Newman and Cragg, 2016; Center for Disease Control, 2019). Since nearly two-thirds of our current antimicrobial drugs are linked to microorganisms (Clardy et al., 2006; Newman and Cragg, 2016), one of our goals was to cultivate a diverse array of bacteria from a non-human mammalian microbiome and screen these isolates for the production of antimicrobial metabolites. Growing microbial populations in the laboratory that are representative of the overall microbial diversity within an inoculum continues to be a major limitation for the field of microbiology. Here, we sought to use a combination of selective medium types and treatments of the inocula that might enrich for certain groups present in the oral and gut microbiomes of a roadkill raccoon. In order to capture a more comprehensive sample of microbial diversity, we used high-throughput sequencing of 16S rRNA gene amplified libraries to characterize the original inocula and the cultivated populations that were washed from the surface of agar media.

We anticipated finding a significantly lower percentage of cultivable organisms relative to the molecular-based measures of diversity from each orifice, which we described as the cultivated percentage. We also anticipated that the identity of recovered taxa would be similar between media types and even orifices. The differences between organisms cultivated on different media, from different orifices was low. This suggested that the original inocula were not as differentially diverse as expected, despite differing measures of richness in the molecular controls. However, when observing the significance described in the permanova and permdisp, the percentage of explained variation was low. This suggested that the stoichiometry of diverse and variable inocula could play a large part in the outcome of this and any similar cultivation experiment, particularly when plating across multiple medium types. Based on the low percentage of explained variation, we also approached this data set from the perspective of the unobserved or residual variation, such that some of this discussion will aim to pose questions about what makes cultivation so difficult. Overall, we observed that cultivation yielded not just a fraction of what was detected in sequence data, but rather expanded the total diversity that potentially described the original sample.

### Cultivation Can be Optimized at Large Scales

Convergence of microbial communities occurred between the mouth and rectum for both the molecular samples and the cultivated communities. This was expected since as an animal undergoes decomposition, the microbial diversity decreases, especially in rectal communities (Guo et al., 2016), and becomes dominated by certain taxa, namely Proteobacteria (Metcalf et al., 2013). It is also important to point out that the preservation of a “typical” oral or rectal community was unlikely to be observed, not only due to changes as decomposition mentioned above, but also that structures may not be intact after collision with a vehicle. In our samples, despite being a single time point roughly 8 h after death, the mouth was dominated by Proteobacteria while the rectum samples contained mostly Firmicutes, Proteobacteria, and Bacteroides (Supplementary Figure 3). Overall, about 87% of the microbial families detected in the molecular controls were shared between the mouth and rectum. This increase in shared taxa was also expected, as the proportion of cultivable organisms was expected to be lower (Jensen, 1968) and the differences in diversity would not be detected through the selection of cultivability. Because of the small proportion of molecularly characterized microorganisms that are routinely recovered in the laboratory, we employed multiple variations in cultivation, like medium type and inoculum treatments to “cast a wider net.”

The treatments that were used selected for organisms that could withstand ethanol stress (e.g., spore-formers), or that were streptomycin resistant. These treatments had a greater effect on the cultivable diversity compared to changes in base medium alone, likely because the base media shared many of the same oxidizable substrates. It is logical to hypothesize that the inoculum exposed to selective treatments would have decreased cultivated richness compared to the same inoculum on an untreated medium of the same type, since the selective agent would inhibit the growth of a portion of the community. Instead, the cultivable richness and diversity varied for the same treatments on different base medium types and between orifices (Figure 1). This could be due to the treatments selecting for specific groups of taxa (i.e., antibiotics) or creating an environment where certain taxa were able to out-compete other taxa on non-selective medium (i.e., low nutrients, increased incubation, and slow growers) (Davis et al., 2005). Specifically, medium amended with catalase or streptomycin and inocula treated with ethanol resulted in cultivated organisms not identified on other plates, or even detected in the molecular controls in some cases (Figure 4). One possible explanation would be that this was due to the inherent stochasticity associated with cultivation (discussed below). Despite this variability, we were able to cultivate roughly 57% of the species richness detected with molecular approaches. Previous studies measuring cultivation success with high-throughput sequencing have reported similar levels of cultivable richness from the skin of toads (∼60%) (Medina et al., 2017) and rumen fluid (23%) (Zehavi et al., 2018). Medina et al. (2017) reported greater variation in cultivability between inocula (different animals of the same species from the same area). Our data showed that more variability in cultivated taxa was observed between selective additions to the medium or treatments of the inocula, and not the inocula (different orifices) (Figure 3). Similar to Zehavi et al. (2018), different medium types and treatments increased the cultivable proportion of the original sample.

### Bioactivity and Phylogenetic Diversity of Isolates

The research reported here was based on previous success of using roadkill mammals as a source of antimicrobial-producing microorganisms (Motley et al., 2017). Our intent was to quantify and broaden the diversity of cultivated bacteria by the addition of various media and treatments. This effort was measured by using plate-wash PCR and SSU rRNA gene sequencing to thoroughly assess the taxa that were cultivated. Simultaneously, colonies were also picked for isolation from replicate plates (*n* = 3), screened for antimicrobial production and identified through SSU rRNA sequencing. Colonies were chosen based on differential morphologies. Overall, 7 of the 238 recovered isolates showed bioactivity against the *already antibiotic-resistant* pathogen panel, resulting in a ∼3% hit rate. Five bioactive isolates were sequenced. Three belonged to the phylum Proteobacteria (class Gammaproteobacteria) and two belonged to the phylum Firmicutes. Two isolates were identified as a *Bacillus* sp. and a *Pseudomonas* sp., both genera known to contain species that produce bioactive compounds (Fuller et al., 1971; Raaijmakers et al., 1997; Hamdache et al., 2011; Sumi et al., 2015; Motley et al., 2017). Interestingly, the isolates identified as an *Enterococcus* sp. and *Klebsiella* sp. showed inhibitory activity to a member of its own genus, *Enterococcus faecium* and *Klebsiella pneumonia*, respectively. This finding was consistent with the hypothesis that closely related organisms often have a negative effect on each other due to resource overlap (Hibbing et al., 2009), which has been demonstrated for these genera (de Vos et al., 2017). In addition, some populations could be making antimicrobial metabolites, like bacteriocins, that are effective against close relatives (Chao and Levin, 1981). Future directions include sequencing the genome of each isolate, investigating biosynthetic gene clusters, and using liquid chromatography and mass spectrometry to investigate putative compounds produced in the bioassays.

### Cultivation Yields Complex Data

The cultivation and isolation of many organisms at once can be incredibly laborious. New technologies and approaches can help increase throughput, which is necessary to overcome the re-isolation of abundant, common, and easy to cultivate organisms. However, it is still important to be able to assess the efficacy of any cultivation effort. Unfortunately, we uncovered an intrinsic property of cultivation that is not readily realized unless assessed at a large scale like it was here, variability. Here, we did not detect the same cells (e.g., colonies measured in PWPCR) consistently growing on replicate plates (Supplementary Figure 4).

Neither differences in cultivation strategy nor orifice sampled could explain the majority of the observed variation in community structure (Figures 5A,B). We can attribute unexplained variance (i.e., residuals) observed between communities (i.e., beta diversity) to stochastic effects of cultivation, but that is not fundamentally measurable in this case. We can speculate that randomness would play a role in cultivation as samples are diluted (Justice et al., 2017; Zehavi et al., 2018) or the spatial heterogeneity of the inoculum is altered, as demonstrated on a much larger scale using leaf litter (Albright et al., 2019). In these instances, we could expect different inocula resulting in different assemblages of cultivated organisms. We also know that microorganisms immobilized on an agar surface are still able to interact through motility or diffusion of metabolites, making certain competitive adaptations more advantageous (Chao and Levin, 1981). In this experiment, the agar media allowed physical separation of individual cells in the inocula and constrained microbial populations to form colonies, potentially forcing interactions with neighboring colonies that could result in competition and inhibition of growth through secondary metabolite production (Chao and Levin, 1981). The randomness of which populations are in close proximity to one another could play a role in which members will thrive from the same inoculum source, affecting the outcome of which taxa are cultivated (Traxler et al., 2013).

Cultivation on agar plates warrants special consideration when discussing diversity. The relative distribution of populations on an agar surface affects their prevalence and the overall composition of the cultivated community. Further, the physiology and colony morphology of each population dictates their prevalence and that of the surrounding populations on an agar surface. Colony size, especially surface area, is impacted by traits such as growth rate, surface motility (i.e., gliding motility, swarming), presence of inhibitory metabolites, and the availability of resources. These differences in biomass distribution were important when deciding how to measure diversity, a critical metric for assessing cultivation on an agar surface. Unlike liquid cultures, varying biomass (i.e., colony size) from different taxa that might produce an equal number of colonies can over-estimate the relative abundance of that taxon, studies relying on relative abundance to characterize diversity should consider this (Medina et al., 2017; Cummings et al., 2020). If a population (A) has more proficient growth and forms a larger colony than population (B), DNA extracted from each population would indicate that taxon A was more abundant in the original inoculum, incorrectly representing the evenness of the original inoculum. This is especially true when relative abundance is calculated based on equimolar DNA concentration during library preparation. To mitigate any biased evenness, species richness and Jaccard distances were used to measure alpha and beta diversity, respectively.

In this data set, about half of the ASVs detected after cultivation were not detected in the molecular controls. This same phenomenon was observed by Zehavi et al. when cultivated rumen OTUs outnumbered the OTUs detected in the original rumen sample and its dilutions (1,012 out of 1,698) (Zehavi et al., 2018). This discrepancy may speak to the power of cultivation relative to the power of direct molecular analyses in describing the diversity of a community. However, both of these approaches come with their own caveats. Cultivation makes assumptions about an organism’s ability to grow in the laboratory, while sequencing is dependent on methodology, sequencing chemistry and sampling depth. In this study, rarefaction curves indicated that sequencing efforts were sufficient (Supplementary Figure 5). One potential explanation for the large proportion of ASVs not detected in the controls could be that in combination with the different media and treatments, we were able to give some of the less abundant microorganisms a growth advantage. For example, members of the bacterial family Wohlfarhtiimonodaceae and members of the yeast family Metaschinikowiceae, were more associated with the addition of streptomycin to media (Table 1), and not detected in the molecular controls (Figure 4). Streptomycin may have selected against the more abundant or more competitive organisms, allowing taxa from these families to grow. Additionally, observing Staphylooccaeae to be significantly associated with the ethanol treatment was unintended, as we anticipated selecting for spore formers. However, this was not surprising as ethanol has been shown to increase biofilm formation in staphylococcal species (Luther et al., 2015). Lastly and in regard to high proportions of ASVs in cultivated samples, systemic contamination and the presence of spurious ASVs from the PBS should be addressed. Similar to Zehavi et al. (2018), we believe this is unlikely, as we observed no growth on the plates incubated with PBS, nor were there any identifiable patterns among more rare taxa that would skew the data. Further, because presence absence was used to describe ASVs, we would anticipate this having a large effect if spurious ASVs were present across all samples, making more rare ASVs have equal weight to abundant ASVs.

Cultivation is critical to answer broad challenges of microbial ecology like deciphering microbial metabolisms or specific challenges like obtaining new isolates for antibiotic discovery. The study of pure cultures remains the best approach to comprehensively describe an organism’s physiological and metabolic properties, and efforts to optimize cultivation strategy have proven successful (Connon and Giovannoni, 2002; Stevenson et al., 2004; Marmann et al., 2014; Lagier et al., 2015; Oberhardt et al., 2015; Yang et al., 2015; Browne et al., 2016; Henson et al., 2016; Berdy et al., 2017; Bartelme et al., 2020). Our data adds to previous studies (Medina et al., 2017; Zehavi et al., 2018) that also sought to assess how medium type and treatment could increase cultivated diversity, but adds the goal of “casting a wider net” to increase the diversity of bioactive isolates. We were able to add to our library of bioactive isolates and show that microbiomes from roadkill mammals are a useful source of bioactivity, as observed in Motley et al. (2017) Lastly, we showed that through a thoughtful cultivation approach driven by bulk molecular analysis of cultivated taxa, we could cultivate a larger proportion of the diversity within an inoculum and even taxa not detected in the molecular controls. This work adds to the growing assessment of cultivation strategies using newer tools, like high-throughput sequencing, and shows how these methods can be applied to drug discovery efforts. Cultivation is influenced by many factors, and this work highlights some of those intricacies, like variability and stochasticity. Cultivation is complex and challenging but cultivating in combination with molecular tools can expand the observed diversity of an environment and its community.

## Data Availability Statement

The datasets presented in this study can be found in online repositories. The names of the repository/repositories and accession number(s) can be found in the article/Supplementary Material.

## Author Contributions

EJ conceived and conducted the experiments, analyzed data, and wrote the manuscript. BS conceived the experiments, analyzed data, and wrote the manuscript. Both authors contributed to the article and approved the submitted version.

## Conflict of Interest

The authors declare that the research was conducted in the absence of any commercial or financial relationships that could be construed as a potential conflict of interest.

## Supplementary Material

Supplementary Figure 1 Bioassay plate showing three isolates generating a zone of inhibition within pathogen overlay.

Supplementary Figure 2 Pie chart of 117 identified isolates at the genus level.

Supplementary Figure 3 Phylum-level characterization of molecular controls, triplicates shown for mouth (RC2M) and rectum (RC2R).

Supplementary Figure 4 Bar chart describing the variability of consistent recovery of ASVs, data subset. Y axis indicates how many replicates of TSA plates in which the top 25 ASV were observed. Color corresponds to orifice.

Supplementary Figure 5 Rarefaction curves showing plateaued sequencing depth of **(A)** cultivation samples and **(B)** molecular controls.

Supplementary Table 1 A quasi-factorial approach using targeted media and treatments for gut microorganisms.
