# Supplementary material for: Using Plate-Wash PCR and High-Throughput Sequencing to Measure Cultivated Diversity for Natural Product Discovery Efforts
Source: Front Microbiol. 2021 Jul 20;12:675798. doi: 10.3389/fmicb.2021.675798 (PMC8329497; doi:10.3389/fmicb.2021.675798)
Supplement: Supplementary Figure 1 — Bioassay plate showing three isolates generating a zone of inhibition within pathogen overlay. [file Data_Sheet_1.zip › Table S1.docx]

| Table S1. A quasi-factorial approach using targeted media and treatments for gut microorganisms. | | | | |
| --- | --- | --- | --- | --- |
|  | **Media** | | | |
| Treatment/ Selection agents | 0.1 TSA | ROXY | 0.25 R2A | Blood Agar |
| None | X | X | X | X |
| Catalase | X | X | X |  |
| Hemin and alpha-ketoglutarate |  | X |  |  |
| 70% ethanol | X |  | X | X |
| Streptomycin | X |  | X | X |
