# Supplementary figures and images for: Using Plate-Wash PCR and High-Throughput Sequencing to Measure Cultivated Diversity for Natural Product Discovery Efforts
Source: Front Microbiol. 2021 Jul 20;12:675798. doi: 10.3389/fmicb.2021.675798 (PMC8329497; doi:10.3389/fmicb.2021.675798)

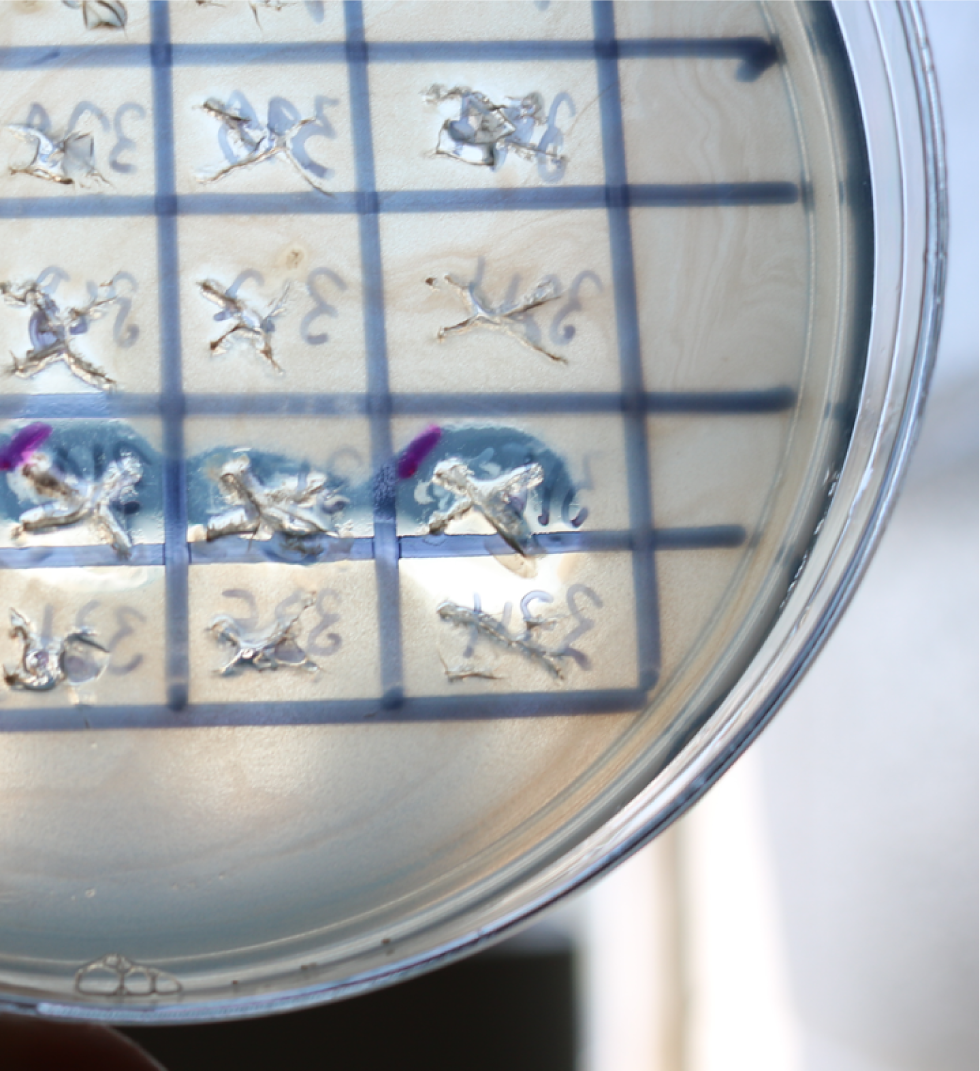

Supplement: Supplementary Figure 1 — Bioassay plate showing three isolates generating a zone of inhibition within pathogen overlay. [file Data_Sheet_1.zip › Figure S1.TIF]

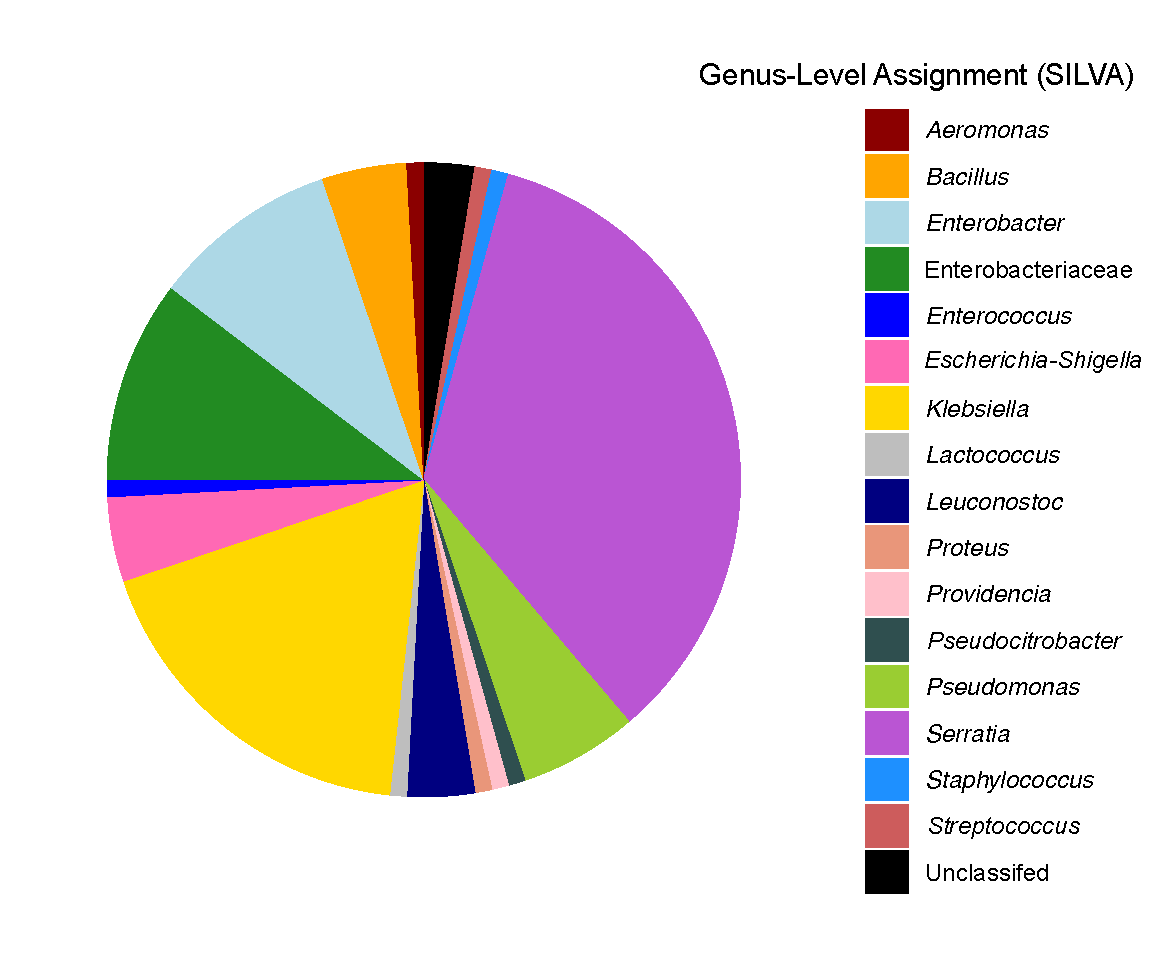

Supplement: Supplementary Figure 1 — Bioassay plate showing three isolates generating a zone of inhibition within pathogen overlay. [file Data_Sheet_1.zip › Figure S2.TIFF]

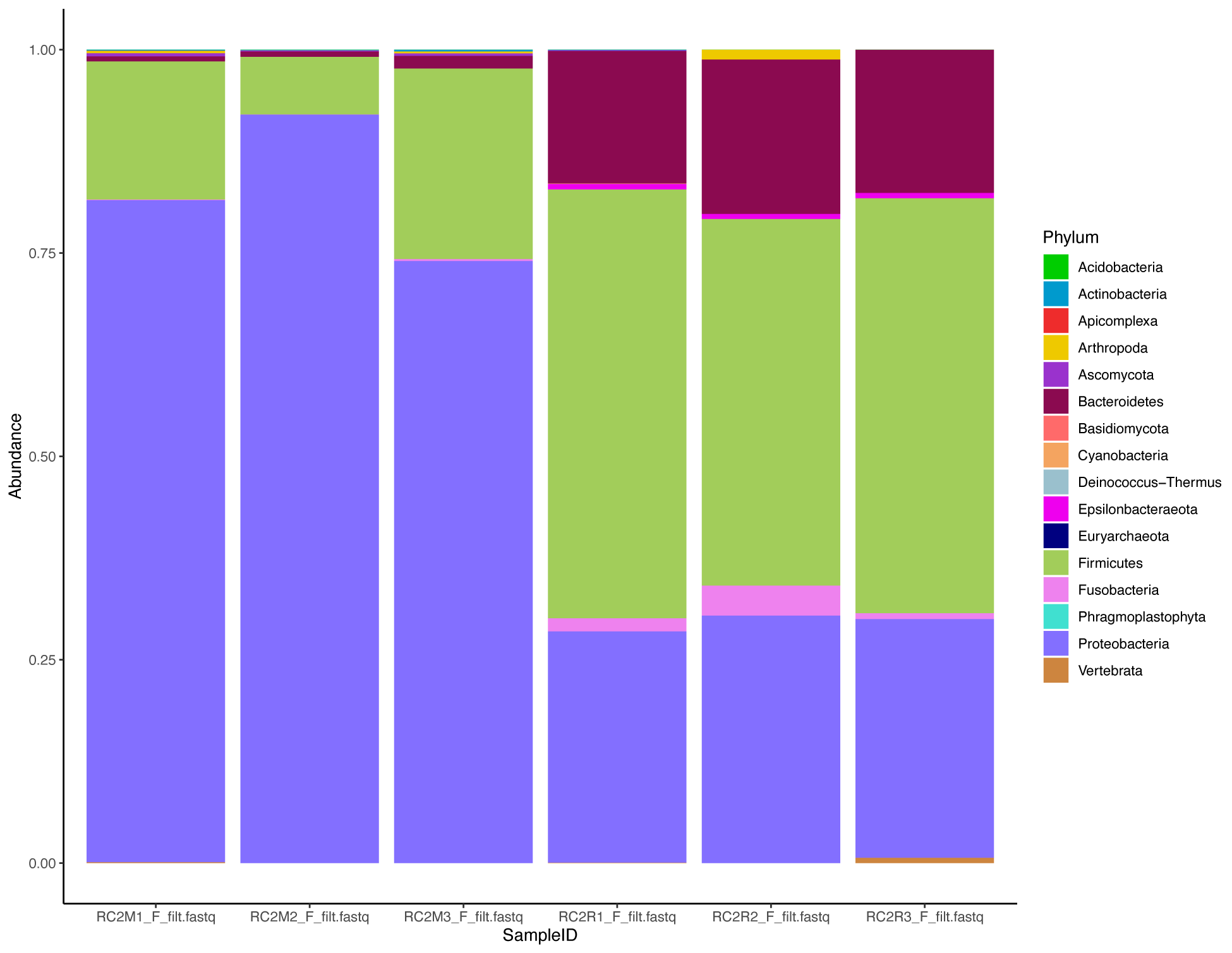

Supplement: Supplementary Figure 1 — Bioassay plate showing three isolates generating a zone of inhibition within pathogen overlay. [file Data_Sheet_1.zip › Figure S3.TIF]

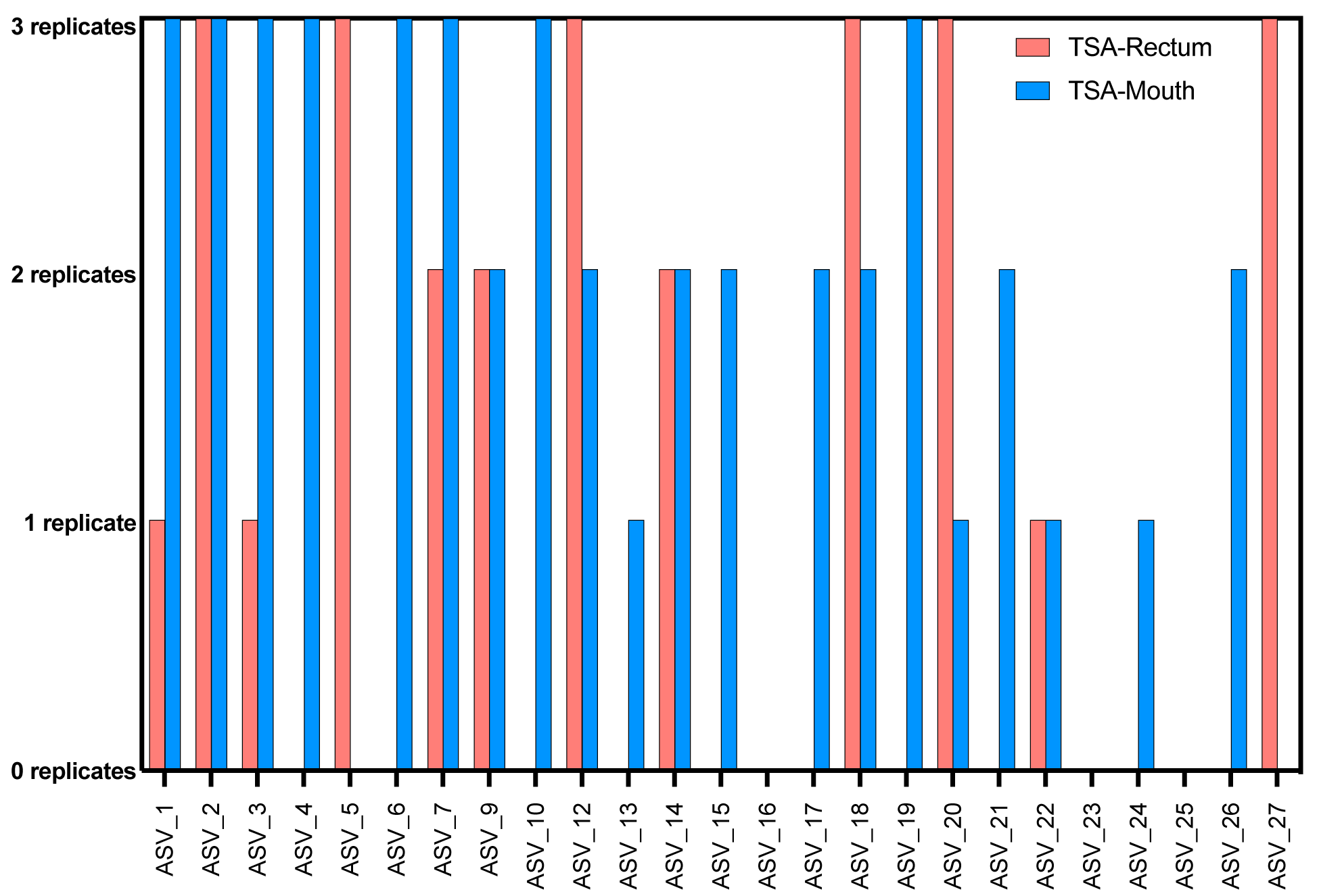

Supplement: Supplementary Figure 1 — Bioassay plate showing three isolates generating a zone of inhibition within pathogen overlay. [file Data_Sheet_1.zip › Figure S4.TIF]

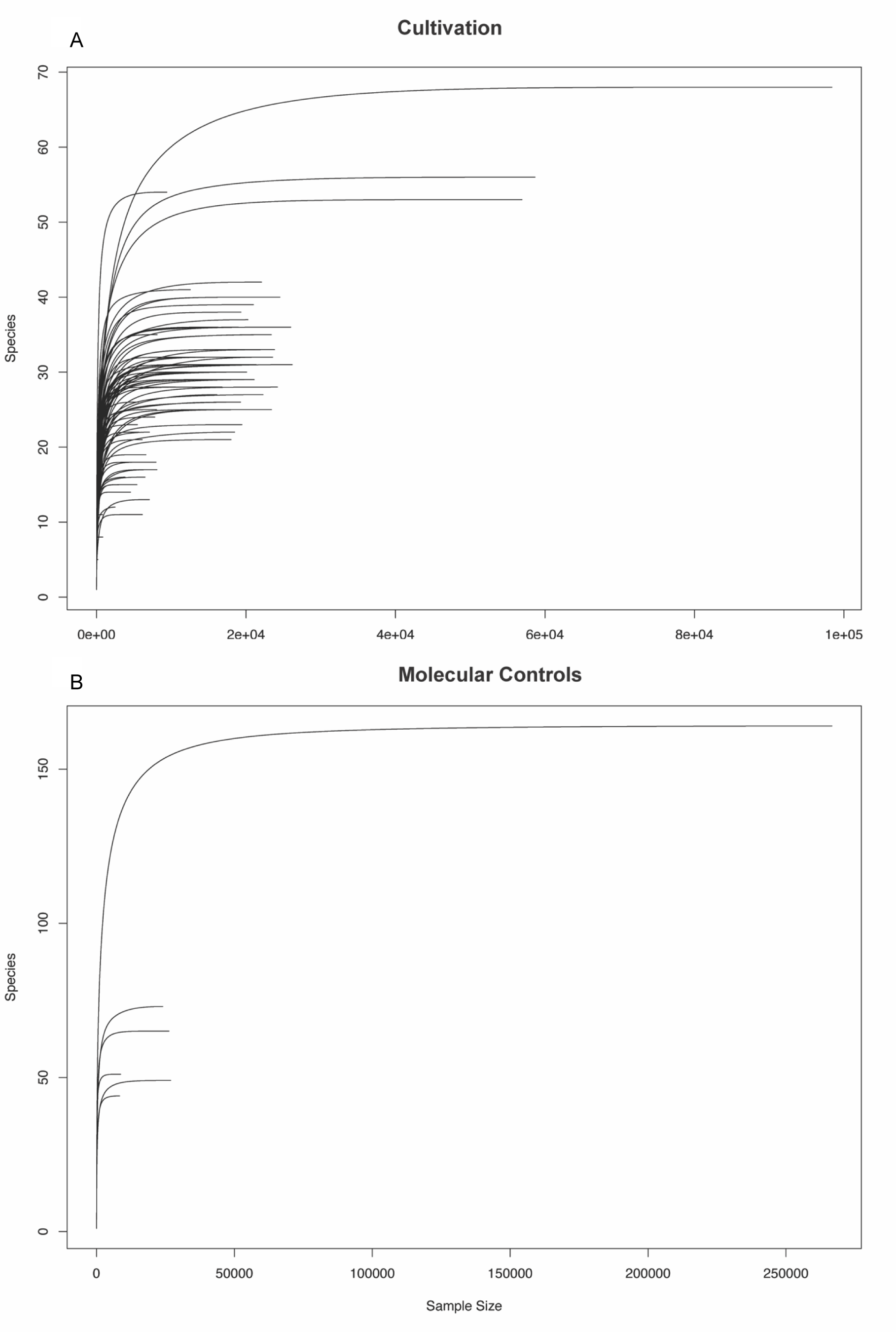

Supplement: Supplementary Figure 1 — Bioassay plate showing three isolates generating a zone of inhibition within pathogen overlay. [file Data_Sheet_1.zip › Figure S5.TIF]
